# Mixed methods analysis of the cultural acceptability and competence of SNAP in Rhode Island and Connecticut

**DOI:** 10.1016/j.pmedr.2026.103532

**Published:** 2026-06-05

**Authors:** Emily Elenio, Alexia Sanchez Contreras, Coco Huang, Elena Hurtado Solberg, Maya Vadiveloo, Alison Tovar

**Affiliations:** a Department of Behavioral and Social Sciences, Brown University School of Public Health, United States; b Brown University, Providence, RI, United States; c Department of Nutrition, University of Rhode Island, United States

**Keywords:** Supplemental nutrition assistance program, Cultural competence, Cultural acceptability, Mixed-methods

## Abstract

**Objective:**

This mixed-method study examined Supplemental Nutrition Assistance Program (SNAP) participants' perceptions of cultural acceptability and competence.

**Methods:**

In Summer 2023 and 2024, 678 and 462 SNAP participants, respectively, living in Rhode Island or Connecticut enrolled in the study. Participants completed an online questionnaire with closed and open-ended questions about perceived cultural barriers. We analyzed closed-ended questions quantitatively to assess the frequency of reported cultural barriers and used content analysis to analyze open-ended responses.

**Results:**

Most participants (95%) reported being able to buy foods that fit within their culture using SNAP benefits and most felt (89%) that their cultural backgrounds were respected when using and enrolling in SNAP. Participants identified grocery stores' wide availability of foods as a facilitator for cultural acceptability. Most participants felt their cultural background is respected when using SNAP. However, among participants reporting cultural barriers, such concerns were intertwined with other structural barriers like cost, lack of transportation, and stigma. Some participants reported that cultural factors were a nonissue as they identified as “lacking culture”.

**Conclusion:**

This study offers an insightful glimpse into how the identities of SNAP participants impact their experience with the program and indicate that most participants perceive SNAP as culturally acceptable and competent.

## Highlights

• Most participants consider SNAP culturally competent and acceptable.
• Wide food availability at grocery stores supports cultural acceptability.
• Cultural barriers include high cost, limited transportation, and stigma.

## 1 Introduction

Food insecurity is a public health concern in the United States and disproportionately impacts historically marginalized racial and ethnic groups (Kim-Mozeleski et al., 2023; Waxman et al., 2022). Social safety net programs like the Supplemental Nutrition Assistance Program (SNAP) are critical for ameliorating food insecurity (Serchen et al., 2022). SNAP serves racially and ethnically diverse populations with recent estimates indicating that 26% of participants identify as Black and 15% as Hispanic (Department of Agriculture et al., 2025). As SNAP increasingly serves communities with distinct cultural backgrounds, understanding how cultural factors shape program experience and outcomes is essential.

Food and mealtimes are widely recognized as integral to culture and identity (Dirks and Hunter, 2012) as cultural preferences shape food choices, culinary practices, and social relationships (Neely et al., 2015). There is growing recognition that culture, particularly cultural acceptability and competence, can affect food security and nutrition security (Briones Alonso et al., 2018; Neely et al., 2014; Wright et al., 2021). Cultural acceptability, sometimes referred to as cultural relevance, considers the influence of cultural food preferences on diet and nutrition and has been cited as a condition for food security (Briones Alonso et al., 2018; Hammelman and Hayes-Conroy, 2015). Cultural competence is rooted in clinical care and reflects interactions between service providers and the people they serve (Greene-Moton and Minkler, 2020; Selig et al., 2006). Although cultural factors shape food insecurity, prevailing measures rarely capture cultural acceptability or competence (Brown et al., 2019).

While the impact of SNAP on food insecurity has been well documented, limited research has explored participants' perspectives on cultural acceptability and competence (Barnes et al., 2023; Gundersen, 2022). Qualitative research studies with few participants (< 15) have identified lack of cultural competence and acceptability as barriers to participation in SNAP and the Special Supplemental Program for Women, Infants and Children (WIC) (Duffy et al., 2022; Louie et al., 2020; Wetherill and Gray, 2015). Assessing cultural factors remains challenging due to the lack of methodological guidance (Joassart-Marcelli et al., 2017) and the deeply personal nature of how individuals and social groups define culture and cultural identity. To better understand participants' perceptions of cultural acceptability and competence when using SNAP, we conducted a mixed-methods study of SNAP participants with children.

## 2 Methods

### 2.1 Study overview

We conducted a secondary analysis of baseline and follow-up data collected in May–September 2023 and June–October 2024 from the What's On Your Kid's Plate Study (WOYPk), which is a part of the larger What's On Your Plate Study (WOYP). Details of both studies are published elsewhere (Tovar et al., 2025) and briefly described here. The WOYP study evaluates a state-wide SNAP fruit and vegetable incentive and assesses changes in fruit and vegetable intake. Participants from the WOYP who reported having at least one child between the ages of 1–8 were invited to participate in the WOYPk, the primary objective of which is to assess children's consumption of fruits and vegetables. The online survey was hosted by Qualtrics and provided in English and Spanish.

Study participants were at least 18 years old, spoke English or Spanish, received SNAP, lived in Rhode Island or Connecticut, reported having access to a phone and email, and were the primary caregiver of a child between 1 and 8 years old. Participants were recruited from community partners and via text blasts to WIC listservs. To detect fraudulent actors, all text-blast participants completed text verification and reCAPTCHA. During high volume periods, participants were randomly called to confirm their zip code and date of birth; all contacted participants successfully completed these verification checks. Participants were invited to complete the follow-up survey in Summer 2024. This study was approved by the institutional review boards at Brown University and the University of Rhode Island and met both institutions' guidelines for the protection of human subjects with regard to safety and privacy.

### 2.2 Participants

The WOYPk survey was completed by 776 participants at baseline and 527 at follow-up.

### 2.3 Measures

Given the lack of validated questions on cultural acceptability and competence of SNAP (Joassart-Marcelli et al., 2017), the study team developed them. Community partners, including Spanish-speaking stakeholders, provided feedback on the questions. Questions were developed in English and then translated by the co-principal investigator (co-PI), a native Spanish speaker. To minimize participant burden and survey fatigue, open-ended questions about cultural acceptability were asked at baseline and questions about cultural competence at follow-up. We anticipated that participants' perceptions of cultural factors were unlikely to change between baseline and follow-up given the relatively short follow-up time (6–12 months) and identical inclusion criteria at both timepoints.

We defined cultural acceptability as the extent to which food purchases with SNAP benefits align with participants' cultural preferences, and cultural competence as the interactions between SNAP participants and program staff. Each construct was assessed with a yes/no question followed by an open-ended prompt asking participants to describe their experience. For cultural acceptability, participants were asked, “Are you able to find foods that fit within your culture using your SNAP dollars?”. For cultural competence, participants were asked, “Do you feel like your cultural background is respected when interacting with individuals that work with SNAP such as caseworkers or grocery store employees?” Participants answering yes were asked to describe how they were able to find culturally relevant foods or felt respected; those answering “no” were asked to describes the barriers or experiences of disrespect they encountered.

### 2.4 Data analysis

We used a mixed-methods approach, specifically an explanatory sequential design in which the survey first asked a close-ended question, followed by an open-ended question.

We analyzed close-ended survey questions and descriptive statistics in R Studio Version 12. All open-ended responses were coded using NVivo 12. Four research assistants (undergraduate and graduate public health students) and the research coordinator, a doctoral public health nutrition student, reviewed responses, created a codebook, met to discuss coding, and iteratively revised the codebook with oversight from both PIs. To assess coding reliability, the research coordinator reviewed all responses coded by the research assistants and identified discrepancies. Disagreements were resolved through discussions with the research assistants, with the full study team consulted when coding decisions had implications for the topic structure. We did not calculate a formal interrater reliability statistic given the iterative process. Responses were excluded if they were illogical or did not relate to the topic. We excluded 102 responses resulting in 678 responses for cultural acceptability and excluded 65 responses resulting in 462 responses for cultural competence.

Given the short length but large volume of open-ended responses, responses were analyzed using qualitative content analysis. We iteratively developed codes, identified potential categories and topics using abstraction. We generated topics, rather than themes, as content analysis typically involves identifying and interpreting the meaning and context of frequently occurring keywords or content within the data resulting in insights that are less robust compared to themes (Hsieh and Shannon, 2005).

## 3 Results

At baseline, most participants received WIC (88.6%) and identified as women (96.0%). Over half of participants were categorized as food insecure (58.1%) (Table 1). Nearly 40% of participants identified as Hispanic and 15% as Black. Most participants reported high cultural acceptability and competence; 95.4% reported that they could find foods that fit within their culture and 89.0% reported that their cultural background was respected by SNAP caseworkers and store employees (Table 2).

**Table 1:** Sociodemographic characteristics of adult Supplemental Nutrition Assistance Program (SNAP) participants with children in Rhode Island and Connecticut, Summer 2023.

Participant characteristics	
Participant characteristics	N=678^1^
Age	32.4 (6.8)
Female	651 (96.0%)
Attended Some College or More	376 (55.5%)
Employed At Least Part-time	321 (47.3%)
Household Receives Special Supplemental Nutrition Program for Women, Infants, and Children (WIC)	601 (88.6%)
Food Insecure	394 (58.1%)
Born Outside of the United States	127 (18.7%)

*Race & Ethnicity*	
Hispanic	263 (38.8%)
Non-Hispanic White	236 (34.8%)
Non-Hispanic Black	105 (15.5%)
Non-Hispanic Multiple Race	50 (7.4%)
Non-Hispanic Asian	15 (2.2%)
Non-Hispanic Other	9 (1.3%)

SNAP: Supplemental Nutrition Assistance Program; WIC: Special Supplemental Nutrition Program for Women, Infants, and Children.

1 Mean (SD) / n (%).

**Table 2:** Perceptions of cultural acceptability and competence with illustrative quotes among adult Supplemental Nutrition Assistance Program (SNAP) participants with children in Rhode Island and Connecticut, Summer 2023 and Summer 2024.

Can you find foods that fit within your culture?		Illustrative Quotes
Supplemental Nutrition Assistance Program (SNAP)	*N* = 678	
Yes	647 (95.4%)	“I haven't had any problem, I always find everything I need in the supermarket, always looking for specials so that it lasts the whole month.” “All of the foods we eat are readily available at our local grocery stores.”
No	31 (4.6%)	“Usually at Walmart they have a section for Hispanic food or a Goya aisle. However, it's very limited.” “I can find what we eat at most supermarkets however I do notice certain items are more pricey than your normal foods.”
Do you feel like your cultural background is respected?		
Supplemental Nutrition Assistance Program (SNAP)	462	
Yes	411 (89.0%)	“I have not encountered anyone who I've had issues with because of my culture. I've gotten spoken to with respect and have been helped without any issues.” “They always find a way to help me to understand everything and I feel okay. Always available to help, my husband does not speak English at all and they always find someone to translate. Overall, they are great people.”
No	51 (11.0%)	“Usually the employees tend to feel that it is ok to assume things about my culture and say unfair unnecessary comments.” “The case worker I feel really look down on my kind of people and I feel if it was up to them they wouldn't give me SNAP.”

SNAP: Supplemental Nutrition Assistance Program.

We identified four major topics, further described below: access to culturally appropriate food, interactions with SNAP workers, negative cultural experience, and perceptions of cultural factors as a non-issue.

**Topic 1: Most participants highlighted the wide availability and ease in finding foods that fit within their culture when using their SNAP dollars.** Many participants expressed how their local supermarkets or corner stores offer culturally relevant products. Some participants explicitly highlighted ethnic supermarkets or ethnic/Hispanic aisles. The wide availability of products at most supermarkets was highlighted as participants noted they can complete their grocery shopping at a single store. While few participants raised concerns about the quality of cultural foods at their local supermarkets, most reported that supermarkets offer a suitable selection of cultural foods.

*“I go to my local Spanish grocery store called [local store name] and they are always stocked with what I need.”*

*“Always, the stores I got [sic] to such as Walmart, corner stores, or Stop & Shop always have what I need.”*

*“I just use it at my local grocery stores such as Stop & Shop. They have the international aisle where I can find some of the things from my cultural background and use my SNAP card when I am checking out for those items.”*

**Topic 2: Participants perceive most interactions with SNAP and grocery store workers as too brief and transactional to result in meaningful exchanges of cultural identity.** Many participants described interactions with SNAP and grocery store workers as brief and straightforward, with most conversations centered on recertification, renewing benefits, or small talk. Some participants mentioned that SNAP workers and grocery store workers do not engage in conversations beyond SNAP. As a result, most participants did not report cultural barriers. Some participants elaborated that interactions mainly consist of yes/no questions leaving little opportunity for cultural exchanges and also negative interactions. A few participants mentioned that they rarely or never interact with SNAP workers, as most of the process can be done online.

*“Very professional in every way. They never ask extra things. They just give me whatever I need and whatever I ask for. It's never about my culture, I've never had a problem.”*

*“They are always helpful and quick. Helpful if there is an issue. Nothing related to culture in how they interact with me.”*

**Topic 3: Negative cultural experiences were tied to intersecting structural barriers.** For the few participants who reported barriers to accessing culturally acceptable foods when using SNAP, they reported a wide variety of challenges; most of them were related to cost and transportation. The high costs of food coupled with low wages were cited as key barriers. Some participants also reported how their ethnic grocery stores do not accept SNAP which further limits their spending potential.

*“I don't have a car to get to the places that sell my cultural food. And if I do happen to be able to get a ride they do not accept food stamps, and I do not make enough money to be able to buy food from there so I just don't buy it.”*

*“When I need to buy Jamaican food here in [town name], there are little to no options. And there are no busses available to take me to the stores.”*

A few participants who reported that their cultural backgrounds were not respected often discussed feelings of stigma due to their race, ethnicity, language, and/or for using SNAP benefits. Notably, some participants expressed that their experience of stigma was tied to their cultural identity, while others mentioned it was tied to their participation in SNAP. At grocery stores, some participants reporting feeling judged when presenting their Electronic Benefits Transfer (EBT) cards at checkout.

*“They make you feel less than them sometimes they be yelling or catching a attitude because you walk-in to asked for help and when they look at my English difficulties since I am Spanish they act like no one can't help me especially because my color”.*

*“They're not too friendly, kind of judge you for applying for help or having a SNAP card.”*

*“Some of the time grocery store employees give different energy when they see a lot of food being purchased or when WIC card or EBT cards are being swiped.”*

**Topic 4: Some participants identify as lacking a culture due to eating Americanized foods.** Some participants indicated that they lacked a culture and thus, did not find accessing culturally acceptable food or interactions with SNAP workers as a barrier. Many of these responses characterized their diets as “normal”. Participants connected American diets to “lacking culture”. While many of these participants identified as non-Hispanic white, there were some racial minorities who reported this sentiment. This may indicate participants' views of their perceived “American” diets and potential acculturation.

*“I don't have a culture. I'm White and eat regular food.”*

*“They always have patience for me. I personally don't feel like I have a cultural background that they can disrespect.”*

## 4 Discussion

Given the changing demographics of SNAP and growing recognition of culture in the food security literature, examining cultural acceptability and competence is increasingly important. We found that most participants did not identify cultural acceptability nor cultural competence as a significant barrier to their SNAP experience. Our mixed-methods findings expand on prior qualitative studies that have identified cultural acceptability and competence as barriers (Duffy et al., 2022; Wetherill and Gray, 2015; Bode et al., 2024) by drawing on a larger sample and integrating multiple methods. Overall, participants reported being able to purchase foods that fit within their culture using SNAP, with the qualitative responses highlighting the importance of wide product availability in stores. These findings likely also reflect that our sample's dietary patterns align with the American food environment and may face fewer cultural barriers. Similarly, participants described interactions while using SNAP benefits as largely positive and brief. However, a small proportion of participants (< ∼10%) reported cultural barriers, most often related to cost and transportation constraints that limited access to cultural foods. For those who reported poor cultural competence, most reported experiences of stigma tied to their cultural background and/or for receiving SNAP. These findings suggest that while cultural barriers are not a predominant concern within this sample of SNAP participants, it is critical to balance culturally informed efforts with initiatives to alleviate structural challenges to improve food security and program satisfaction.

In our sample, the wide availability of grocery stores, coupled with international aisles and ethnic grocery stores were key facilitators for cultural acceptability. Participants often specified Hispanic products (e.g., Goya), specific stores, and/or international isles to describe how they find foods that fit within their culture. Even participants who reported shopping at large chains, rather than ethnic grocery stores, expressed that the selection was sufficient to address their cultural needs. Rather than contradicting prior qualitative studies that have identified access to culturally relevant foods as a barrier for SNAP participants (Louie et al., 2020; Wetherill and Gray, 2015), our findings likely reflect differences in sample and setting. The aforementioned qualitative studies were conducted within specific populations (e.g., Asian and Pacific Islanders in Los Angeles) and/or settings (e.g., farmers markets that accept SNAP). For instance, Wetherhill and others (Wetherill and Gray, 2015) found that African-American participants reported poor cultural acceptability of food items available at farmers markets in Oklahoma (Wetherill and Gray, 2015). By contrast, our sample resides in Rhode Island and Connecticut, two small densely populated Northeastern states, and approximately 40% of participants identified as Hispanic, a group whose food preferences are comparatively well represented in regional grocery offerings. Our finding align with broader American retail trends where the number of grocery store items per store has grown from 7000 in the 1990s to 40,000 items by the 2010s (Ruhlman, 2017). However, increased availability does not necessarily equate to improved access. SNAP participants live on average within 1.88 miles of a grocery store but typically traveled 3.26 miles to shop, suggesting that price, quality, and selection, not distance alone, shape purchasing decisions (Ploeg et al., 2015). Overall, these findings suggest that perceived cultural acceptability reflects a combination of regional retail density, sample composition, and alignment between participants' food preferences and the food environment.

Our findings suggest that most SNAP participants in our sample feel their cultural background is respected when using their SNAP benefits. Interactions with caseworkers were generally described as brief and transactional, differing from prior studies that identified poor cultural competence as a barrier to enrollment and participation (Louie et al., 2020; Greene et al., 2023; Larimore, 2018). This divergence may reflect broader program shifts, including the rise of online grocery shopping and digital recertification processes that reduce opportunities for cultural exchange (Rummo et al., 2022).

A minority of participants reported experiencing cultural barriers, often compounded by structural barriers such as lack of transportation, poor access to food, and high cost. These experiences align with research showing that ethnic minority groups face disproportionate barriers particularly cost, transportation, and stigma (Louie et al., 2020; Johnson-Green and Claflin, 2021; Wood and Horner, 2016). Prior research has established that many SNAP participants feel judged when using their SNAP benefits (Blau et al., 2025) and that stigma is associated with a greater risk of food insecurity (Hatton et al., 2024). While our study did not directly assess stigma, discrimination and cultural competence are interrelated and some of our participants connected their perceptions of cultural competence to stigma. For this subset of participants, cultural barriers, stigma, and structural barriers appear to intersect and exacerbate existing challenges, including transportation barriers and heightened feelings of marginalization when seeking food assistance. It is also possible that cultural barriers may be most salient for populations whose cultural food practices are not well represented in the American food environment.

Growing virtual options for SNAP re-enrollment and grocery shopping may reduce stigma and transportation barriers. In our data, some participants who reported stigma felt that presenting an EBT card at checkout elicits stigmatizing responses from grocery staff, whereas prior research suggests that online grocery platforms may reduce stigma by concealing of EBT use (Hatton et al., 2024; Kim et al., 2019; Vedovato et al., 2022). However, associated costs with online fees and reduced social interaction highlight that digitalization is not a panacea (Kim et al., 2019; Vedovato et al., 2022). Addressing cultural competence in tandem with structural barriers remains critical to improving equity and satisfaction in SNAP.

The qualitative responses indicated that some participants perceived a “lack of culture” and thus did not report cultural barriers when using SNAP. Some participants described themselves as having no distinct culture and justifying this by noting how they eat “plain American food”. These responses offer valuable insights into the identities of some SNAP participants and how they relate to the foods they consume and their cultural self-perception. Open-ended responses of having “no culture” raise important concerns, as food and culture are deeply linked with senses of belonging and most critically, personal identity (Dirks and Hunter, 2012; Williams-Forson and Walker, 2012) and warrants careful interpretation. Rather than indicating an absence of cultural identity, these responses may reflect how cultural acceptability, as a construct, is most salient to those whose food practices fall outside dominant American patterns. Participants whose diets align with the US food environment may not perceive “culture” as a meaningful lens for evaluating SNAP because the program already accommodates their preferences by default. This perception may also reflect participants' views of American food, possibly symbolizing the acculturation processes, especially among non-US born participants. Moreover, such perceptions could illustrate how American food functions as a symbol of globalization, influencing participants' relationships with food and culture. The globalization of food systems and the wide availability of food products can blur cultural distinctions, making “American food”, both a local and global category. Understanding these nuanced identities is essential, as cultural food preferences strongly shape food purchasing and consumption behaviors, yet they may be evolving or complex among diverse SNAP recipients.

There are some important limitations to note. First, this study relied on self-reported data through an online survey platform, and it is possible that some participants did not understand the survey questions which were not pre-tested with participants. Second, open-ended survey questions are unlikely to yield as rich of insights compared to qualitative interviews or focus groups. Third, our sample primarily reflects women (96.0%) and Hispanic participants (40%), rather than non-Hispanic Asian participants and Indigenous participants who were underrepresented. Further, participants were from Rhode Island and Connecticut, two smaller and densely populated Northeastern states, and thus, these findings may have differed for other populations such as rural communities and immigrant populations. Future that includes more geographically and ethnically diverse samples is warranted. This study has several strengths: First, the large sample size allowed for quantitative analysis. Our analysis offers insights regarding cultural acceptability and competence of SNAP from over 500 participants, which is meaningful given the limited literature. Secondly, this study offers a novel approach of using mixed methods to investigating cultural concepts on a large scale.

## 5 Conclusion

Among this sizable sample, we found that SNAP participants from Rhode Island and Connecticut perceived SNAP as largely culturally acceptable and competent. Our findings, drawn from a larger mixed-method study sample, differ from prior qualitative studies that identified poor cultural competence as a barrier to enrollment and participation, suggesting that cultural experiences may vary across contexts and populations. While global evidence underscores the importance of cultural tailoring (Briones Alonso et al., 2018), our results indicate that cultural barriers were not predominant. Nonetheless, poor cultural acceptability and competence may remain significant barriers for other groups, such as non-Hispanic Asians, Pacific Islanders, and Indigenous populations. SNAP-Education (SNAP-Ed), which provides nutrition education and obesity prevention programming, has historically provided culturally tailored nutrition education, including materials adapted for diverse linguistic and cultural communities (Greene et al., 2023; Datz, 2025). The planned elimination of SNAP-Ed, under the “One Big Beautiful Bill Act” raises important questions about how cultural tailoring will be supported moving forward (Datz, 2025). Our results indicate that while culturally informed efforts remain important, greater emphasis on addressing structural barriers like as transportation, cost, and stigma may further strengthen food security and program satisfaction for participants in Rhode Island and Connecticut. Whether these priorities hold for SNAP participants in other regions, particularly those whose cultural food practices are less represented in the US food environment, remains an important question for future research.

## CRediT authorship contribution statement

**Emily Elenio:** Writing – original draft, Project administration, Methodology, Investigation, Formal analysis, Data curation, Conceptualization. **Alexia Sanchez Contreras:** Writing – review & editing, Investigation, Formal analysis. **Coco Huang:** Writing – review & editing, Investigation, Formal analysis. **Elena Hurtado Solberg:** Writing – review & editing, Investigation, Formal analysis. **Maya Vadiveloo:** Writing – review & editing, Supervision, Project administration, Funding acquisition, Data curation, Conceptualization. **Alison Tovar:** Writing – review & editing, Supervision, Project administration, Funding acquisition, Data curation, Conceptualization.

## Funding sources

This work is funded by the U.S. Food Policy Evaluation Program at the University of Illinois Chicago, which is supported by a grant (2020–85774) from Bloomberg Philanthropies' Food Policy Program (www.bloomberg.org). The contents of this publication do not necessarily reflect the views or policies of Bloomberg Philanthropies. Bloomberg Philanthropies was not involved in design and conduct of the study; collection, management, analysis, and interpretation of the data; preparation, review, or approval of the manuscript; or decision to submit the manuscript for publication. MKV received funding from the K01 Career Development award from the National Heart, Lung, and Blood Institute (5K01HL165104).

## Declaration of competing interest

The authors declare that they have no known competing financial interests or personal relationships that could have appeared to influence the work reported in this paper.

## Data Availability

The authors do not have permission to share data.
